# Kidney Injury Molecule‐1 Expression in Pathological T1b Clear Cell Renal Cell Carcinoma: A Putative Biomarker of High Immune‐Inflamed Status and Recurrence

**DOI:** 10.1111/pin.70024

**Published:** 2025-05-14

**Authors:** Ayuna Sugai, Kosuke Miyai, Keiichi Ito, Susumu Matsukuma, Kimiya Sato

**Affiliations:** ^1^ Department of Basic Pathology National Defense Medical College Tokorozawa Japan; ^2^ Department of Laboratory Medicine National Defense Medical College Hospital, National Defense Medical College Tokorozawa Japan; ^3^ Department of Urology National Defense Medical College Tokorozawa Japan

**Keywords:** cancer‐specific survival, clear cell renal cell carcinoma, immunohistochemistry, kidney injury molecule‐1, recurrence, tumor‐associated immune cell

## Abstract

Kidney injury molecule‐1 (KIM‐1) is a potential prognostic marker of advanced‐stage clear cell renal cell carcinoma (ccRCC) and is associated with tumor immunogenicity. Little is known about its role in early‐stage ccRCC, especially in pathological T1b (pT1b) disease, which shows a higher recurrence rate than pT1a disease. Resected specimens from 112 pT1b ccRCC cases were reviewed and immunohistochemically analyzed for KIM‐1 expression. High membranous KIM‐1 expression was defined as H score ≥ 140, based on the immunoreactive intensity and area, and cytoplasmic expression in ≥ 10% of cancer cells was considered as high cytoplasmic KIM‐1 expression. KIM‐1 expression status was compared with clinicopathological variables, including tumor‐associated immune cell (TAIC) status. Among the 112 cases, high membranous and cytoplasmic KIM‐1 expression was observed in 30 (27%) and 38 (34%) cases, respectively. High membranous KIM‐1 expression was significantly associated with a higher nuclear grade, tumor necrosis, hot TAIC status, and shorter recurrence‐free survival (RFS) and cancer‐specific survival, whereas high cytoplasmic expression was only related to a higher nuclear grade. Multivariate Cox regression analysis revealed that high membranous KIM‐1 expression and tumor necrosis were independent predictors of shorter RFS. Our results indicate that membranous KIM‐1 expression could be a biomarker for predicting postnephrectomy recurrence in pT1b ccRCC.

## Graphical abstract

High membranous kidney injury molecule‐1 (KIM‐1) expression in pathological T1b (pT1b) clear cell renal cell carcinoma (ccRCC) was significantly associated with a higher nuclear grade, tumor necrosis, hot tumor associated immune cell (TAIC) status, and shorter recurrence‐free survival (RFS) and cancer‐specific survival, and was an independent predictor of shorter RFS. High membranous KIM‐1 expression is associated with *high immune‐inflamed status* and could be a useful biomarker for predicting postnephrectomy recurrence in pT1b ccRCC.

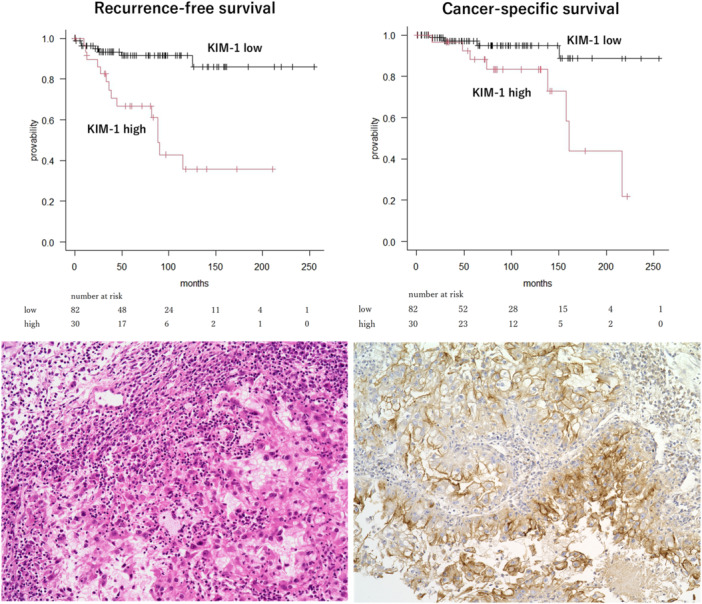

**Abbreviations**
ccRCC: clear cell renal cell carcinoma
CSS: cancer‐specific survival
HAV: hepatitis A virus
H&E: hematoxylin and eosin
ICI: immune checkpoint inhibitor
ISUP: International Society of Urological Pathology
KIM‐1: kidney injury molecule‐1
LVI: lymphovascular invasion
PD‐1: programmed cell death‐1
PD‐L1: programmed cell death‐ligand 1
pT1: pathological T1
RFS: recurrent free survival
STAT‐3: signal transducer and activator of transcription 3
TAIC: tumor‐associated immune cell
TIM‐1: T cell immunoglobulin and mucin domain 1
WHO: World Health Organization

## 1 Introduction

Clear cell renal cell carcinoma (ccRCC) is the most common subtype of renal cell cancer (RCC), accounting for approximately 75% of all RCC cases [1]. Although patients with unresectable, metastatic, or recurrent RCC have poor prognoses, the recent development of immune checkpoint inhibitors (ICIs), such as programmed cell death‐1 (PD‐1) and programmed cell death‐ligand 1 (PD‐L1) inhibitors, as well as antivascular endothelial growth factor‐targeted therapy have improved their outcomes [2, 3]. Localized RCCs, particularly pathological T1 (pT1) tumors, generally exhibit favorable patient outcomes and are best managed with nephron‐sparing surgeries such as partial nephrectomy [4, 5]. However, previous studies have revealed that patients with pT1b RCC have significantly lower 5‐year cancer‐specific survival (CSS) and recurrence‐free survival (RFS) rates than those with pT1a RCC, indicating the need for prognostic biomarkers using surgical specimens in these patients with heterogeneous outcomes [6]. Although the primary postoperative factors for tumor recurrence in early‐stage ccRCC include tumor necrosis and microvascular invasion, the predictive biomarkers remain poorly elucidated [7, 8].

Kidney injury molecule‐1 (KIM‐1), a type 1 cell membrane glycoprotein, is highly expressed in the regenerative epithelial cells of the proximal tubules and has been widely recognized as a biomarker for acute kidney injury [9]. Cuadros et al. [10] reported that KIM‐1 activates the signal transducer and activator of transcription 3 (STAT‐3) pathway and promotes the expression of growth/angiogenic factors by inducing interleukin‐6 expression, which is likely to promote tumor growth and metastasis. Recently, KIM‐1 was reported to be a useful serum marker for early detection of localized RCCs and metastatic/recurrent tumors [11, 12]. Another study showed that high levels of serum KIM‐1 in patients with RCC were associated with adverse pathological findings and short metastasis‐free survival and overall survival [12]. Although high serum KIM‐1 levels have been reported to be useful for detecting pT1a tumors, their significance as a prognostic marker in localized tumors, especially pT1b tumors, has not been fully investigated [12].

Blockade of immune checkpoint components, such as PD‐1/PD‐L1, has shown considerable oncological benefits and has shifted treatment strategies targeted at RCC [13, 14, 15]. The immune‐inflamed status of tumors is evaluated on the basis of the immunoreactivity of PD‐1/PD‐L1 in inflammatory and tumor cells as well as histological evaluations, including the presence of tumor‐associated immune cells (TAICs) and tumor‐infiltrating lymphocytes, which are related to ICI efficacy and poor patient outcomes [16]. A recent subanalysis of a randomized, double‐blind clinical study using atezolizumab for adjuvant therapy of ccRCC (IMmotion010) revealed that a higher serum KIM‐1 level was a risk factor for poor prognosis, but improved clinical outcomes in the atezolizumab versus placebo group, indicating its association with high tumor immunogenicity [17]. Although the biological interactions between immune cells and tumor cells expressing KIM‐1 are worth investigating, few studies have examined KIM‐1 expression in ccRCC tumor cells. Han et al. [18] reported that KIM‐1 immunoreactivity in ccRCC was higher than that in the normal kidney; however, its relationship with tumor progression and patient outcomes was not evaluated in their study. Additionally, no previous study has attempted to relate immunohistochemical KIM‐1 expression in ccRCC to tumor immune‐inflamed status.

In the present study, we histologically reviewed sequentially resected specimens from 112 ccRCC cases categorized as pT1b (clinical N0M0). Immunohistochemical analysis for KIM‐1 was performed to determine whether (1) high KIM‐1 expression is a common finding in localized ccRCCs; (2) the status of KIM‐1 expression is correlated with clinicopathological variables, especially TAIC status, as a histological marker of tumor immune‐inflamed status; and (3) high KIM‐1 expression affects RFS and CSS.

## 2 Materials and Methods

### 2.1 Ethical Approval and Consent to Participate

This study was performed in accordance with the Declaration of Helsinki and approved by the Ethics Committee of the National Defense Medical College (registration number: 5156). Informed consent was obtained from all participants using an opt‐out form published on our website. Patients were given the opportunity to decline to provide their clinical records and paraffin‐embedded slides of the surgical specimens used for research.

### 2.2 Case Enrollment

One hundred and twelve patients with pT1b (clinical N0M0) ccRCC who underwent partial or complete nephrectomy at the National Defense Medical College Hospital between 2000 and 2022 were included in this study. Clinical information of the patients was obtained from the medical records. The median follow‐up period for the 112 patients was 78 months (range: 0.1–256 months). All patients were postoperatively evaluated for local recurrence/distant metastasis every 3–6 months for the first 5 years and every 6–12 months thereafter. Follow‐up examinations included a physical examination, chest radiography, abdominal/chest computed tomography, and blood tests.

### 2.3 Histological Evaluation

**Figure 1:**
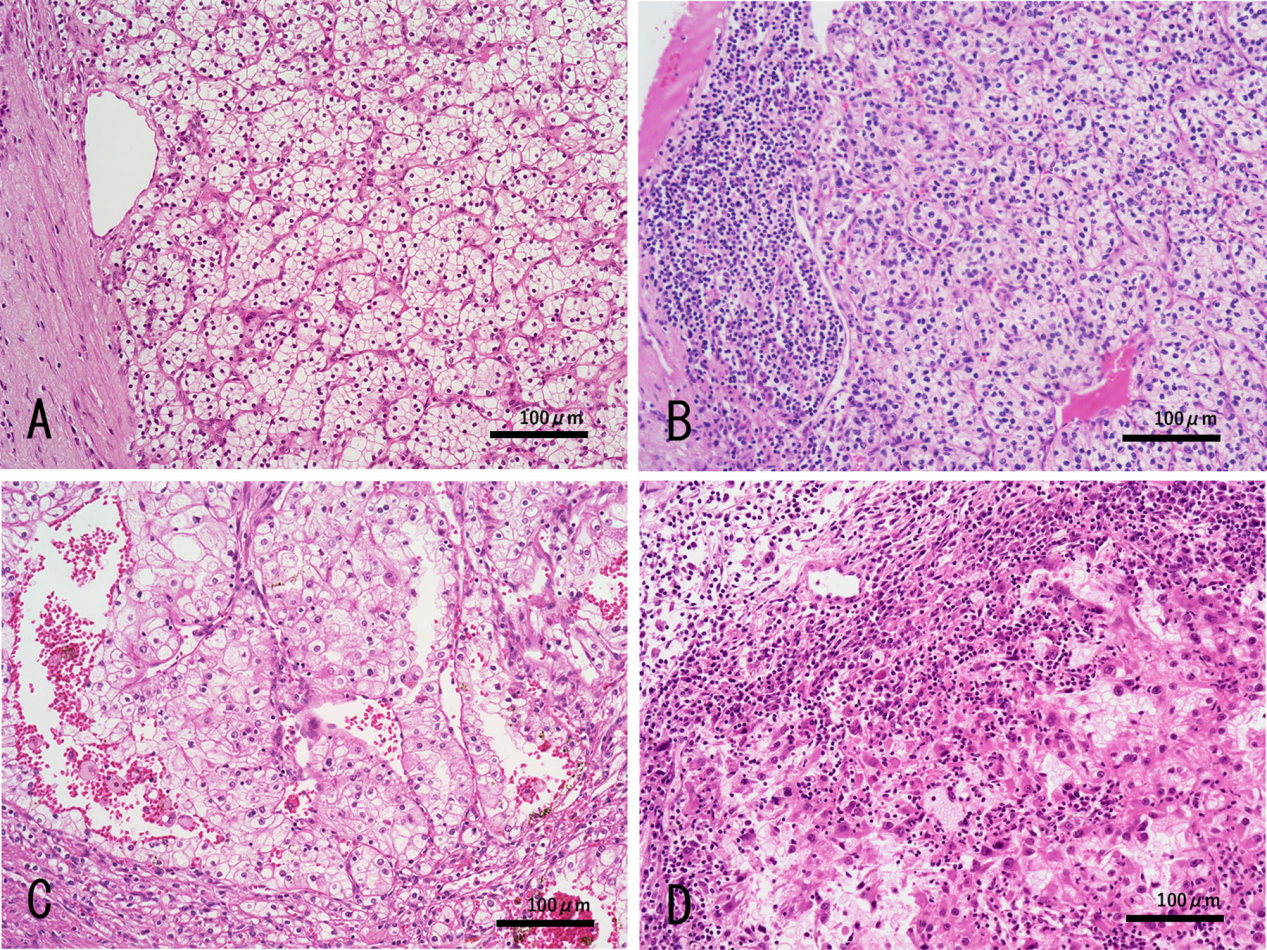
Representative images of tumor‐associated immune cell (TAIC) status. Panels exhibiting TAICs: (A) none (desert, intensity 0); (B) diffuse and high peritumoral TAICs (excluded, intensity 2); (C) focal and low intratumoral TAICs (inflamed, intensity 1); and (D) diffuse and high intratumoral TAICs (inflamed, intensity 2). Case (D) was designated as “hot” TAIC status. Hematoxylin and eosin staining (original magnification, ×200).

Histological slides of the 112 ccRCC samples were retrieved from the Department of Laboratory Medicine at the National Defense Medical College Hospital. An experienced genitourinary pathologist (K.M.) reviewed all of the hematoxylin and eosin (H&E)‐stained slides of the specimens to confirm the pathological findings according to the latest World Health Organization (WHO) criteria (5th edition, 2022) [19]. The pT categorization of the disease was performed according to the eighth edition of the American Joint Committee on Cancer Staging Manual [20]. Tumor nuclear grading was evaluated according to the WHO/International Society of Urological Pathology (ISUP) nuclear grading system [21, 22]. Tumor necrosis was defined as coagulative “dirty” necrosis characterized by abundant cell debris and neutrophilic aggregation, which did not include degenerative “ghost‐like” changes [23]. Sarcomatoid/rhabdoid changes and lymphovascular invasion (LVI) of cancer cells were tabulated as present or absent. Histologically, TAICs were evaluated on the basis of the intensity and location of mononuclear and granulocytic infiltration on H&E‐stained slides [16]. The TAIC intensities were classified into the following three categories: none (intensity 0), focal/low (intensity 1), or diffuse/high (intensity 2). “Diffuse/high” intensity was defined as more than 50 immune cells per x400 field in more than 50% of the whole tumor area, and other patterns of immune cell infiltration were defined as “focal/low” intensity. The locations of the TAICs were also classified into three groups: none (desert), peritumoral (excluded), or intratumoral (inflamed). “Peritumoral” area was defined as within a range of 2 mm from the tumor‐stromal borderline. Using combinations of these scores, cases with TAIC intensity score 2 and intratumoral location were designated as showing “hot” TAIC status and others were categorized as showing “not hot” TAIC status [16]. Representative images of the TAIC classifications are shown in Figure 1.

### 2.4 Immunohistochemical Assessments

Each one representative block of the lesions was cut into 4‐μm‐thick sections and subjected to immunohistochemical analysis. Deparaffinized sections were subjected to autoclave antigen retrieval with 0.01 mol/L citrate buffer (pH 6.0) at 120°C for 10 min. Endogenous peroxidase was blocked by treatment with 5% hydrogen peroxide for 5 min, and nonspecific binding was blocked by treatment with 2% goat serum for 10 min. The sections were then incubated with 1:100 dilution of goat polyclonal antibody against KIM‐1 (AF1750; R&D Systems) at 4°C overnight. The slides were incubated with a dextran polymer reagent combined with secondary antibodies and peroxidase (Dako) for 30 min at room temperature. Specific antigen‐antibody reactions were visualized with 0.2% diaminobenzidine tetrahydrochloride and hydrogen peroxide, and counterstaining was performed with Mayer's hematoxylin. The resected ccRCC specimen, including the damaged peritumoral renal tubules, was used as the positive control.

**Figure 2:**
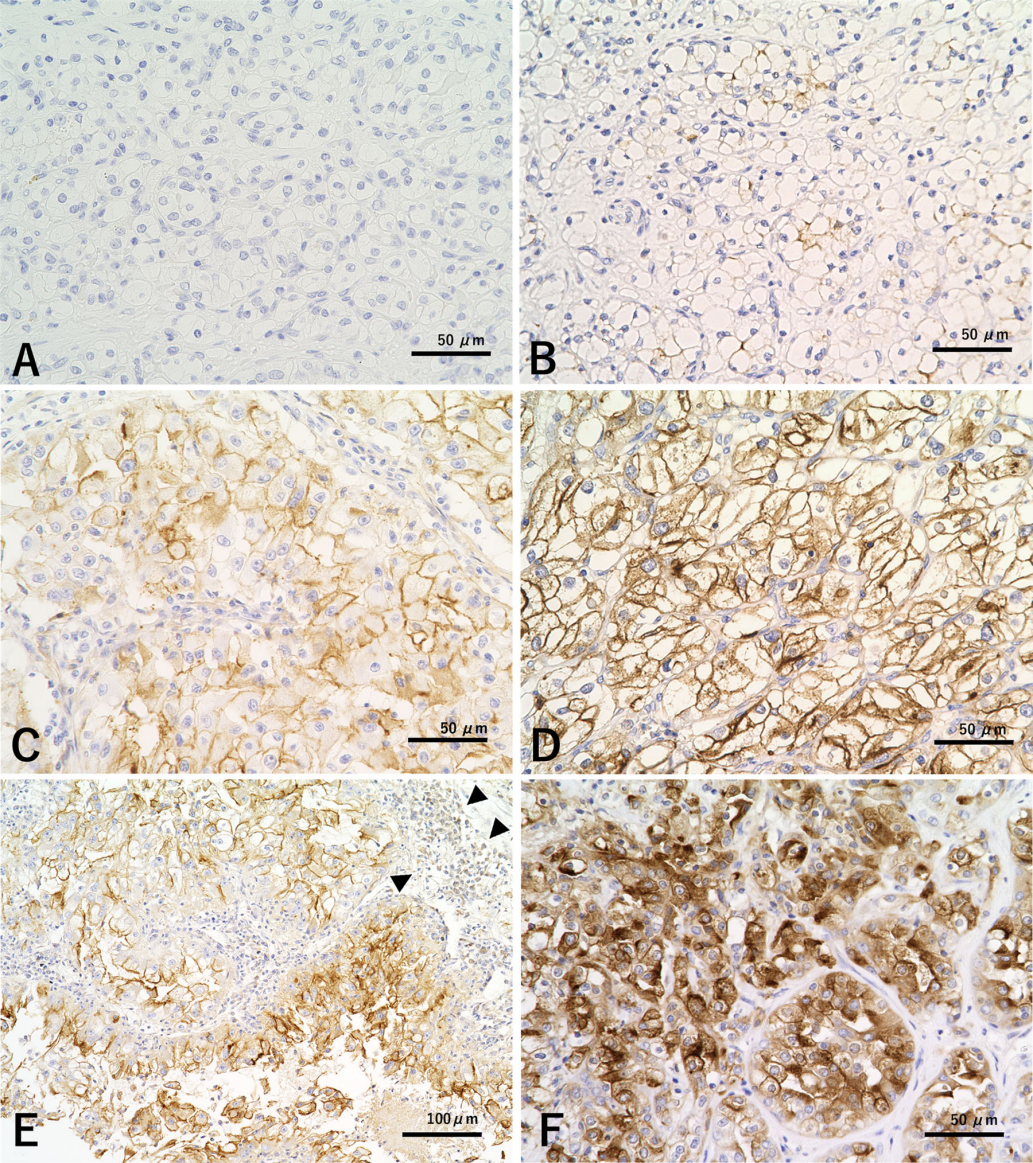
Immunohistochemical findings for kidney injury molecule‐1 (KIM‐1) expression in clear cell renal cell carcinoma (ccRCC). Tumor cells showing (A) no staining (score 0) and (B) weak (score 1), (C) moderate (score 2), and (D) strong (score 3) membranous expression. (E) A case showing strong membranous KIM‐1 expression and hot tumor‐associated immune cell status. Some immune cells are also immunoreactive for KIM‐1 (arrowheads). (F) A ccRCC case showing cytoplasmic KIM‐1 expression as well as moderate membranous expression. Immunoperoxidase staining (original magnification, ×400 for (A)–(D) and (F); ×200 for (E)).

Membranous and cytoplasmic immunoreactivity in an entire tumor area of each representative slide was evaluated for assessing KIM‐1 expression. According to the scoring system used in previous reports, membranous KIM‐1 immunoreactivity was assessed using the H score, which is based on the staining intensity and fraction of positive tumor cells [24, 25, 26]. The intensities of membranous KIM‐1 immunoreactivity were classified into the following four categories: no staining (score 0), weak (score 1), moderate (score 2), or strong (score 3) and multiplied by the percentage of positive cells, resulting in H scores ranging from 0 to 300 [24]. Using receiver operating characteristic curves based on RFS and CSS, a cutoff value of H score was set as 140. For statistical analysis, H score ≥ 140 and < 140 were considered to indicate high and low membranous KIM‐1 expression, respectively [25, 26]. Cytoplasmic expression of KIM‐1, regardless of the degree, was also evaluated; immunoreactivity of ≥ 10% and < 10% in all cancer cells was considered to indicate high and low cytoplasmic KIM‐1 expression, respectively. The scoring in each case was performed by a genitourinary pathologist (K.M.). Representative cases of membranous and cytoplasmic KIM‐1 expression are shown in Figure 2.

### 2.5 Statistical Analysis

Statistical analyses were performed using EZR version 1.68 (Saitama Medical Center, Jichi Medical University, Saitama, Japan), a graphical user interface for R software (version 4.0.5; R Core Team and Foundation for Statistical Computing). Clinicopathological parameters were compared between patients with high and low KIM‐1 expression using Fisher's exact test. Differences of KIM‐1 expression status between groups stratified by clinicopathological factors were evaluated by Mann–Whitney *U* test. RFS and CSS were determined using the Kaplan–Meier method, and comparisons were made using the log‐rank test. A Cox proportional hazards regression analysis was used to determine the effects of KIM‐1 expression and other clinicopathological variables on RFS and CSS. Differences were considered statistically significant at *p* < 0.05.

## 3 Results

A histological review confirmed that the tumors in all examined cases were pT1b ccRCCs. In the assessment of the WHO/ISUP grade, 7 (6%), 37 (33%), 49 (44%), and 19 (17%) patients were classified into grades 1, 2, 3, and 4, respectively. Among the 112 cases, 24 (21%) were categorized as showing TAIC intensity score 2, and 21 (19%) showed hot TAIC status. Tumor necrosis was detected in 25 (22%) cases. Only three (3%) cases exhibited sarcomatoid changes. Although two (67%) of the three cases showed high membranous KIM‐1 expression, for statistical reasons (i.e., a small number of cases), sarcomatoid changes were not evaluated in the subsequent analyses. None of the examined cases showed rhabdoid changes in cancer cells. The H score for membranous KIM‐1 expression ranged from 0 to 230 (median, 80), and high membranous KIM‐1 expression (H score ≥ 140) was found in 30 (27%) cases. High cytoplasmic KIM‐1 expression (≥ 10% of cancer cells) was found in 38 (34%) cases. A statistically significant relationship was observed between high membranous and cytoplasmic KIM‐1 expression (*p* < 0.001). A subset of immune cells was also immunoreactive for KIM‐1 (Figure 2E).

### 3.1 Relationship Between KIM‐1 Expression and Clinicopathological Variables

The clinicopathological parameters and membranous KIM‐1 expression status of the examined cases are summarized in Table 1. Cases with low and high membranous KIM‐1 expression showed no significant differences in age (≥ 70 and < 70 years), gender, tumor laterality, or the presence of LVI. Forty (49%) and 28 (93%) cases with low and high membranous KIM‐1 expression, respectively, were categorized as WHO/ISUP grade ≥ 3, with the two groups showing a statistically significant difference (*p* < 0.001). The frequency of tumor necrosis was significantly higher in cases with high membranous KIM‐1 expression than in those with low membranous KIM‐1 expression (53% vs. 11%; *p* < 0.001). Hot TAIC status was significantly more frequent in cases with high membranous KIM‐1 expression than in those with low membranous KIM‐1 expression (*p* = 0.002). The differences in median H scores of membranous KIM‐1 expression stratified by clinicopathological parameters also showed similar results, as shown in Table 2.

**Table 1:** Relationship between membranous KIM‐1 expression and clinicopathological parameters.

	Membranous KIM‐1 expression		
Variables	Low (*n* = 82)	High (*n* = 30)	*p* value
Age (≥ 70/ < 70 years)	25/57	12/18	0.370
Gender (male/female)	60/22	22/8	1.000
Tumor size (> 4–< 5 cm/≥ 5–≤ 7 cm)	41/41	10/20	0.137
Laterality (left/right)	43/39	16/14	1.000
ECOG PS (0/1 and 2)	70/12	26/4	1.000
WHO/ISUP grade (1 and 2/3 and 4)	42/40	2/28	< 0.001
TAIC intensity 2 (%)	10 (12)	14 (47)	< 0.001
Hot TAIC status (%)	9 (11)	12 (40)	0.002
Tumor necrosis (%)	9 (11)	16 (53)	< 0.001
Lymphovascular invasion (%)	33 (40)	17 (57)	0.138

Abbreviations: ECOG PS, Eastern Cooperative Oncology Group Performance Status; KIM‐1, kidney injury molecule‐1; TAIC, tumor‐associated immune cell; WHO/ISUP, World Health Organization/International Society of Urological Pathology.

**Table 2:** Differences in membranous KIM‐1 H scores between groups stratified by clinicopathological variables.

Variables	Median of H scores	*p* value
Age (≥ 70/ < 70 years)	95/70	0.507
Gender (male/female)	90/65	0.521
Tumor size (> 4–< 5 cm/≥ 5–≤ 7 cm)	80/105	0.210
Laterality (left/right)	95/50	0.278
ECOG PS (0/1 and 2)	80/85	0.412
WHO/ISUP grade (1 and 2/3 and 4)	40/118	< 0.001
TAIC intensity (0 and 1/2)	65/155	< 0.001
TAIC status (others/hot)	70/150	0.003
Tumor necrosis (absent/present)	70/150	0.002
Lymphovascular invasion (absent/present)	75/98	0.235

Abbreviations: ECOG PS, Eastern Cooperative Oncology Group Performance Status; KIM‐1, kidney injury molecule‐1; TAIC, tumor‐associated immune cell; WHO/ISUP, World Health Organization/International Society of Urological Pathology.

The clinicopathological parameters and cytoplasmic KIM‐1 expression status of the examined cases are summarized in Table S1. Seventy‐four (66%) and 38 (34%) cases with low and high cytoplasmic KIM‐1 expression, respectively, were categorized as WHO/ISUP grade ≥ 3, with a statistically significant difference (*p* = 0.024). No significant differences were observed in the other parameters.

### 3.2 RFS and CSS Stratified by KIM‐1 Expression

**Figure 3:**
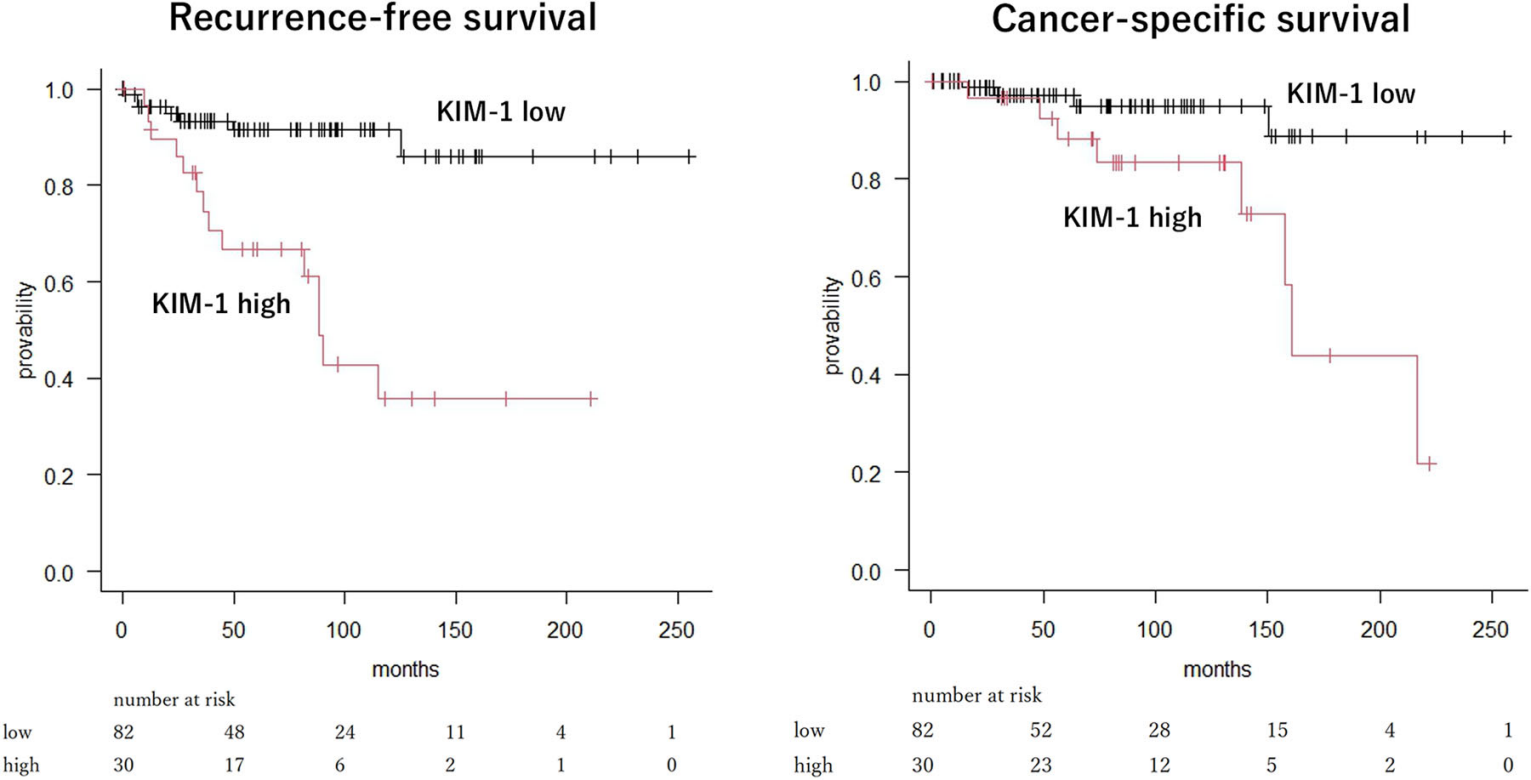
Impact of membranous kidney injury molecule‐1 (KIM‐1) expression on recurrence‐free survival (RFS) and cancer‐specific survival (CSS) in clear cell renal cell carcinoma. Low membranous KIM‐1 expression (black line) versus high membranous KIM‐1 expression (red line): RFS, *p* < 0.001 and CSS, *p* = 0.005.

The 5‐year RFS and CSS rates were, respectively, 67% and 88% in patients with high membranous KIM‐1 expression and 91% and 97% in those with low membranous KIM‐1 expression. The RFS and CSS curves for the 112 patients with pT1b ccRCC stratified by membranous KIM‐1 expression are presented in Figure 3. High membranous KIM‐1 expression was significantly associated with shorter RFS and CSS based on the log‐rank test (*p* < 0.001 and *p* = 0.005, respectively).

For RFS, univariate Cox regression analysis indicated that high membranous KIM‐1 expression, high cytoplasmic KIM‐1 expression, WHO/ISUP grade ≥ 3, hot TAIC status, LVI, and tumor necrosis were predictors of recurrence risk (Table 3a). To avoid multicollinearity between high membranous and cytoplasmic KIM‐1 expression, the former, which showed higher hazard ratio than the latter in the univariate analysis, was used in the multivariate analysis. Subsequent multivariate Cox regression analysis identified high membranous KIM‐1 expression (*p* = 0.044, hazard ratio = 2.88) and tumor necrosis (*p* = 0.021, hazard ratio = 4.19) as independent predictors of short RFS (Table 3b).

**Table 3:** Cox proportional hazards regression analysis estimates of the significance of predictive factors for recurrence‐free survival.

Variables	*p* value	HR (95% CI)
(a) *Univariate Cox regression analysis*		
Membranous KIM‐1 H score ≥ 140	< 0.001	5.77 (2.32–14.32)
Cytoplasmic KIM‐1 expression ≥ 10%	0.037	2.49 (1.06–5.88)
Age ≥ 70	0.921	1.05 (0.41–2.71)
WHO/ISUP grade ≥ 3	0.014	4.67 (1.37–15.91)
Male gender	0.824	1.12 (0.41–3.06)
Right‐side laterality	0.153	1.91 (0.79–4.61)
Hot TAIC status	0.010	3.19 (1.32–7.73)
Lymphovascular invasion	0.034	2.67 (1.08–6.63)
Tumor necrosis	< 0.001	5.85 (2.46–13.91)
(b) *Multivariate Cox regression analysis*		
Membranous KIM‐1 H score ≥ 140	0.044	2.88 (1.03–8.08)
WHO/ISUP grade ≥ 3	0.816	1.19 (0.28–5.06)
Hot TAIC status	0.540	0.70 (0.22–2.22)
Lymphovascular invasion	0.085	2.39 (0.89–6.45)
Tumor necrosis	0.021	4.19 (1.24–14.17)

Abbreviations: CI, confidence interval; HR, hazard ratio; KIM‐1, kidney injury molecule‐1; TAIC, tumor‐associated immune cell; WHO/ISUP, World Health Organization/International Society of Urological Pathology.

Univariate Cox regression analysis showed that high membranous KIM‐1 expression, WHO/ISUP grade ≥ 3, hot TAIC status, LVI, and tumor necrosis were correlated with lower CSS (Table 4a). Multivariate Cox regression analysis, including these five variables, failed to reveal an independent predictor of a short CSS (Table 4b).

**Table 4:** Cox proportional hazards regression analysis estimates of the significance of predictive factors for cancer‐specific survival.

Variables	*p* value	HR (95% CI)
(a) *Univariate Cox regression analysis*		
Membranous KIM‐1 H score ≥ 140	0.011	4.74 (1.43–15.78)
Cytoplasmic KIM‐1 expression ≥ 10%	0.050	3.43 (1.00–11.74)
Age ≥ 70	0.472	1.69 (0.41–6.97)
WHO/ISUP grade ≥ 3	0.024	10.67 (1.36–83.77)
Male gender	0.979	1.02 (0.27–3.77)
Right‐side laterality	0.638	1.32 (0.41–4.24)
Hot TAIC status	0.012	4.31 (1.38–13.47)
Lymphovascular invasion	0.039	3.98 (1.07–14.74)
Tumor necrosis	< 0.001	9.59 (2.54–36.22)
(b) *Multivariate Cox regression analysis*		
Membranous KIM‐1 H score ≥ 140	0.626	1.40 (0.36–5.36)
WHO/ISUP grade ≥ 3	0.435	2.69 (0.22–32.22)
Hot TAIC status	0.925	0.93 (0.20–4.23)
Lymphovascular invasion	0.323	2.07 (0.49–8.79)
Tumor necrosis	0.065	5.50 (0.90–33.63)

Abbreviations: CI, confidence interval; HR, hazard ratio; KIM‐1, kidney injury molecule‐1; TAIC, tumor‐associated immune cell; WHO/ISUP, World Health Organization/International Society of Urological Pathology.

## 4 Discussion

Risk stratification based on postoperative specimens is of utmost importance for the management of pT1b ccRCC because these tumors have significantly lower postoperative 5‐year CSS and RFS rates than pT1a ccRCC [4, 5, 6]. In the present study, 108 (96%) pT1b ccRCC cases showed membranous KIM‐1 expression of any intensity, while 30 (27%) cases showed high membranous KIM‐1 expression (H score ≥ 140). High membranous KIM‐1 expression was significantly associated with WHO/ISUP grade ≥ 3, the presence of tumor necrosis, and hot TAIC status. The log‐rank test results indicated that patients with high membranous KIM‐1 expression had significantly shorter RFS and CSS than those with low membranous KIM‐1 expression, and Cox regression analysis showed that high membranous KIM‐1 expression and tumor necrosis were independent predictors of shorter RFS. Although high cytoplasmic KIM‐1 expression was also associated with WHO/ISUP grade ≥ 3, its prognostic impact on RFS and CSS was less than that of membranous KIM‐1 expression.

Although KIM‐1 is known to be a type 1 cell membrane glycoprotein, previous studies have reported substantial intracellular pools of endogenous KIM‐1, which may participate in the process leading to the degradation of specific target proteins, such as NUR77, a nuclear receptor implicated in the cellular response to injury [27, 28]. In addition, renal cancer cell lines have shown a large cytoplasmic pool of KIM‐1 as well as high expression of KIM‐1 on the cell surface [27]. Cuadros et al. [29] investigated immunohistochemical membranous and cytoplasmic KIM‐1 expression in 98 ccRCCs and reported that high cytoplasmic KIM‐1 expression, not high membranous expression, was correlated with a higher WHO/ISUP grade, stage, and risk group based on the University of California Los Angeles‐Union Internationale Contrele Cancer nomogram. In addition to the differences in the antibodies used in the two studies (goat polyclonal antibody in our study vs. mouse monoclonal antibody in their study) as well as differences in the immunohistochemistry protocols and the criteria for defining cytoplasmic overexpression (i.e., they assessed cytoplasmic expression using the H score), this discrepancy may also be attributable to differences in the case cohorts; their study included 45 (46%) cases with pT2 or higher ccRCCs and 11 (11%) cases with metastatic disease. In fact, in their study, increased cytoplasmic expression was mainly found in advanced and metastatic tumors, and the membranous KIM‐1 expression levels in these tumors were similar to those observed in localized tumors [29]. The association between cellular localization of KIM‐1 and ccRCC progression is also of great interest, since it may explain the different clinicopathological significances of KIM‐1 expression at each developmental stage of ccRCC.

KIM‐1, also known as T cell immunoglobulin and mucin domain 1 (TIM‐1), is expressed on CD4 (+) T cells, plays an important role in regulating the Th2 reaction, and is involved in hepatitis A virus (HAV) autoimmunity, immune tolerance, atopic diseases, and other autoimmune diseases [28]. In patients with systemic lupus erythematosus, increased expression levels of KIM‐1/TIM‐1 mRNA in peripheral blood mononuclear cells have been observed and correlated with disease activity [30]. Another report indicated that HAV infection may protect individuals from atopic diseases by carrying a particular variant of the gene encoding KIM‐1, which could lead to the deletion of certain lymphocyte subsets, such as Th2 cells, or reduce Th2‐cell differentiation [31]. RCC is a highly immunogenic tumor type, and immune cells play an important role in both the progression and therapeutic interventions targeting RCC [32, 33]. Although a randomized, double‐blind, multicenter, phase 3 trial conducted in 215 centers in 28 countries (IMmotion10) showed no significant prognostic improvement with adjuvant atezolizumab after RCC resection [33], a subanalysis indicated that high serum KIM‐1 levels were associated with improved clinical outcomes in patients administered adjuvant atezolizumab, which may indicate a relationship between KIM‐1 expression and immunogenicity in RCC [17]. In the present study, high membranous KIM‐1 expression was strongly associated with hot TAIC status, supporting the aforementioned considerations. In contrast, no significant relationship was observed between cytoplasmic KIM‐1 expression and TAIC status. Interestingly, a subset of peritumoral immune cells exhibited KIM‐1 immunoreactivity (Figure 2E); however, it is not included in the scope and delimitation of the present study. Further investigations are needed to elucidate the mechanisms and functions of KIM‐1 in tumor immune‐inflamed status.

Several previous studies have investigated the serum/urinary levels of KIM‐1 in patients with ccRCC to improve the implementation of treatment strategies, including early detection and risk stratification of diseases [11, 12, 18, 34]. Using plasma samples from the European Prospective Investigation into Cancer and Nutrition, Scelo et al. [11] reported that serum KIM‐1 levels could predict RCC incidence up to 5 years before diagnosis and were associated with a higher risk of death. A similar result was obtained from a recent subanalysis of a multinational prospective study, which indicated that serum KIM‐1 level could be used as a biomarker for early detection of pT1a ccRCCs [12]. In a long‐term follow‐up of another cohort including pT1‐4 RCC cases, higher prenephrectomy plasma KIM‐1 levels were associated with worse metastasis‐free survival and overall survival [12]. Xu et al. [34] investigated the postnephrectomy serum levels of KIM‐1 in 418 patients with RCCs, including 329 patients with ccRCCs (89% had pT2 or higher disease), and indicated that higher serum KIM‐1 levels were associated with worse disease‐free survival and overall survival. In addition, our data suggest that even in cases of localized pT1b, KIM‐1 could be a useful marker for tumor recurrence. Han et al. [18] reported that urinary levels of KIM‐1 were correlated with immunohistochemical KIM‐1 expression in tumor cells but cautioned that serum/urinary KIM‐1 levels may increase due to kidney injury for various reasons, leading to false‐positive results in tumor screening. Therefore, the immunohistochemical assessment of KIM‐1 expression in resected tumors may be superior for predicting tumor recurrence than the measurement of serum/urinary KIM‐1.

The present study had several limitations. First, this was a retrospective study that included a relatively small number of patients from a single institution. Further studies with larger numbers of patients are required to confirm our results. Second, the relationship between KIM‐1 expression in cancer cells and the efficacy of immunotherapy was not investigated. Therefore, a detailed evaluation of tumor immunogenicity has not yet been conducted. Third, we failed to collect serum and/or urinary samples from ccRCC patients; therefore, the correlation between serum/urine levels and the immunohistochemical expression of KIM‐1 was difficult to evaluate.

In conclusion, our studies targeting pT1b ccRCC demonstrated that high membranous KIM‐1 expression was correlated with several adverse clinicopathological variables and hot TAIC status, and was also an independent predictor of shorter RFS. These findings indicate a relationship between KIM‐1 expression and high immune‐inflamed status of tumors, and they suggest that membranous KIM‐1 expression may be a reliable biomarker for postnephrectomy recurrence of pT1b disease.

## Author Contributions

Ayuna Sugai acquired and analyzed data, wrote the initial draft of the manuscript. Kosuke Miyai concepted and designed the study, analyzed data, and critical review of the manuscript. Keiichi Ito, Susumu Matsukuma, and Kimiya Sato assisted with the critical review of the manuscript and agreed to be accountable for the integrity of the work.

## Conflicts of Interest

The authors declare no conflicts of interest.

## Supporting information

Supporting information.

## Data Availability

The data that support the findings of this study are available on request from the corresponding author. The data are not publicly available due to privacy or ethical restrictions.

## References

[1] X. Feng , L. Zhang , W. Tu , and S. Cang , “Frequency, Incidence and Survival Outcomes of Clear Cell Renal Cell Carcinoma in the United States From 1973 to 2014: A SEER‐Based Analysis,” Medicine 98 (2019): e16684. 31374051 10.1097/MD.0000000000016684 PMC6708618

[2] Z. Zhu , Y. Jin , J. Zhou , et al., “PD1/PD‐L1 Blockade in Clear Cell Renal Cell Carcinoma: Mechanistic Insights, Clinical Efficacy, and Future Perspectives,” Molecular Cancer 23 (2024): 146. 39014460 10.1186/s12943-024-02059-y PMC11251344

[3] W. K. Rathmell , R. B. Rumble , P. J. Van Veldhuizen , et al., “Management of Metastatic Clear Cell Renal Cell Carcinoma: ASCO Guideline,” Journal of Clinical Oncology 40 (2022): 2957–2995. 35728020 10.1200/JCO.22.00868

[4] S. Joniau , K. V. Eeckt , S. J. Srirangam , and H. Van Poppel , “Outcome of Nephron‐Sparing Surgery for T1b Renal Cell Carcinoma,” BJU International 103 (2009): 1344–1348. 19040528 10.1111/j.1464-410X.2008.08230.x

[5] B. Ljungberg , L. Albiges , Y. Abu‐Ghanem , et al., “European Association of Urology Guidelines on Renal Cell Carcinoma: The 2022 Update,” European Urology 82 (2022): 399–410. 35346519 10.1016/j.eururo.2022.03.006

[6] Z. L. Zhang , W. Chen , Y. H. Li , et al., “Stage T1N0M0 Renal Cell Carcinoma: The Prognosis in Asian Patients,” Chinese Journal of Cancer 30 (2011): 772–778. 22035858 10.5732/cjc.011.10085 PMC4013300

[7] K. Ito , K. Seguchi , H. Shimazaki , et al., “Tumor Necrosis Is a Strong Predictor for Recurrence in Patients With Pathological T1a Renal Cell Carcinoma,” Oncology Letters 9 (2015): 125–130. 25435945 10.3892/ol.2014.2670 PMC4246637

[8] J. M. Kim , P. H. Song , H. T. Kim , and T. C. Park , “The Prognostic Factors for Patients With pT1a Renal Cell Carcinoma,” Korean Journal of Urology 51 (2010): 233–238. 20428424 10.4111/kju.2010.51.4.233 PMC2858860

[9] T. Ichimura , J. V. Bonventre , V. Bailly , et al., “Kidney Injury Molecule‐1 (KIM‐1), a Putative Epithelial Cell Adhesion Molecule Containing a Novel Immunoglobulin Domain, Is Up‐Regulated in Renal Cells After Injury,” Journal of Biological Chemistry 273 (1998): 4135–4142. 9461608 10.1074/jbc.273.7.4135

[10] T. Cuadros , E. Trilla , E. Sarró , et al., “HAVCR/KIM‐1 Activates the IL‐6/STAT‐3 Pathway in Clear Cell Renal Cell Carcinoma and Determines Tumor Progression and Patient Outcome,” Cancer Research 74 (2014): 1416–1428. 24390735 10.1158/0008-5472.CAN-13-1671

[11] G. Scelo , D. C. Muller , E. Riboli , et al., “KIM‐1 as a Blood‐Based Marker for Early Detection of Kidney Cancer: A Prospective Nested Case‐Control Study,” Clinical Cancer Research 24 (2018): 5594–5601. 30037816 10.1158/1078-0432.CCR-18-1496 PMC6239904

[12] W. Xu , V. Gaborieau , S. M. Niman , et al., “Plasma Kidney Injury Molecule‐1 for Preoperative Prediction of Renal Cell Carcinoma Versus Benign Renal Masses, and Association With Clinical Outcomes,” Journal of Clinical Oncology 42 (2024): 2691–2701. 38701382 10.1200/JCO.23.00699 PMC11539753

[13] Y. S. Hah and K. C. Koo , “Immunology and Immunotherapeutic Approaches for Advanced Renal Cell Carcinoma: A Comprehensive Review,” International Journal of Molecular Sciences 22 (2021): 4452. 33923219 10.3390/ijms22094452 PMC8123195

[14] C. Mazza , B. Escudier , and L. Albiges , “Nivolumab in Renal Cell Carcinoma: Latest Evidence and Clinical Potential,” Therapeutic Advances in Medical Oncology 9 (2017): 171–181. 28344662 10.1177/1758834016679942 PMC5349425

[15] T. K. Choueiri , T. Powles , M. Burotto , et al., “Nivolumab Plus Cabozantinib Versus Sunitinib for Advanced Renal‐Cell Carcinoma,” New England Journal of Medicine 384 (2021): 829–841. 33657295 10.1056/NEJMoa2026982 PMC8436591

[16] C. Ohe , T. Yoshida , J. Ikeda , et al., “Histologic‐Based Tumor‐Associated Immune Cells Status in Clear Cell Renal Cell Carcinoma Correlates With Gene Signatures Related to Cancer Immunity and Clinical Outcomes,” Biomedicines 10 (2022): 323. 35203532 10.3390/biomedicines10020323 PMC8869140

[17] A. Laurence , B. Axel , S. Cristina , et al., “Circulating Kidney Injury Molecule‐1 Biomarker Analysis in IMmotion010: A Randomized Phase 3 Study of Adjuvant Atezolizumab vs Placebo in Patients With Renal Cell Carcinoma at Increased Risk of Recurrence After Resection,” Journal of Clinical Oncology 42 (2024): 4506.

[18] W. K. Han , A. Alinani , C. L. Wu , et al., “Human Kidney Injury Molecule‐1 Is a Tissue and Urinary Tumor Marker of Renal Cell Carcinoma,” Journal of the American Society of Nephrology 16 (2005): 1126–1134. 15744000 10.1681/ASN.2004070530 PMC1307501

[19] P. R. Goswami , G. Singh , T. Patel , and R. Dave , “The WHO 2022 Classification of Renal Neoplasms (5th Edition): Salient Updates,” Cureus 16 (2024): e58470. 38765391 10.7759/cureus.58470 PMC11100973

[20] B. I. Rini , J. M. McKiernan , S. Chang , et al., “Kidney,” in AJCC Cancer Staging Manual (8th ed.), eds. M. B. Amin , S. B. Edge , R. K. Brookland , et al. (Springer, 2017), 739–748.

[21] B. Delahunt , J. C. Cheville , G. Martignoni , et al., “The International Society of Urological Pathology (ISUP) Grading System for Renal Cell Carcinoma and Other Prognostic Parameters,” American Journal of Surgical Pathology 37 (2013): 1490–1504. 24025520 10.1097/PAS.0b013e318299f0fb

[22] P. H. Tan , J. Cheville , R. H. Giles , et al., “Clear Cell Renal Cell Carcinoma,” in WHO Classification of Tumours, 5th edition. Urinary and Male Genital Tumours, ed. The WHO Classification Editorial Board (IARC Press, 2022), 38–42.

[23] T. Kuroe , R. Watanabe , M. Kojima , et al., “Evaluation of the Morphological Features and Unfavorable Prognostic Impact of Dirty Necrosis in Renal Cell Carcinoma,” Journal of Cancer Research and Clinical Oncology 147 (2021): 1089–1100. 33475860 10.1007/s00432-020-03505-2 PMC11801861

[24] K. Bang , J. Cheon , Y. S. Park , et al., “Association Between HER2 Heterogeneity and Clinical Outcomes of HER2‐Positive Gastric Cancer Patients Treated With Trastuzumab,” Gastric Cancer 25 (2022): 794–803. 35524883 10.1007/s10120-022-01298-6

[25] H. L. Kristoffersen , R. Røge , and S. Nielsen , “Comparison of Antibodies to Detect Uroplakin in Urothelial Carcinomas,” Applied Immunohistochemistry & Molecular Morphology 30 (2022): 326–332. 35510771 10.1097/PAI.0000000000001021 PMC9067085

[26] C. Kuempers , T. Jagomast , R. Krupar , et al., “Delta‐Like Protein 3 Expression in Paired Chemonaive and Chemorelapsed Small Cell Lung Cancer Samples,” Frontiers in Medicine 8 (2021): 734901. 34692726 10.3389/fmed.2021.734901 PMC8531433

[27] S. Balasubramanian , S. K. Kota , V. K. Kuchroo , B. D. Humphreys , and T. B. Strom , “TIM Family Proteins Promote the Lysosomal Degradation of the Nuclear Receptor NUR77,” Science Signaling 5 (2012): ra90. 23233528 10.1126/scisignal.2003200 PMC3767312

[28] J. Song , J. Yu , G. W. Prayogo , et al., “Understanding Kidney Injury Molecule 1: A Novel Immune Factor in Kidney Pathophysiology,” American Journal of Translational Research 11 (2019): 1219–1229. 30972157 PMC6456506

[29] T. Cuadros , E. Trilla , M. R. Vilà , et al., “Hepatitis A Virus Cellular Receptor 1/Kidney Injury Molecule‐1 Is a Susceptibility Gene for Clear Cell Renal Cell Carcinoma and Hepatitis A Virus Cellular Receptor/Kidney Injury Molecule‐1 Ectodomain Shedding a Predictive Biomarker of Tumour Progression,” European Journal of Cancer 49 (2013): 2034–2047. 23352434 10.1016/j.ejca.2012.12.020

[30] Y. Wang , J. Meng , X. Wang , et al., “Expression of Human TIM‐1 and TIM‐3 on Lymphocytes From Systemic Lupus Erythematosus Patients,” Scandinavian Journal of Immunology 67 (2008): 63–70. 18052965 10.1111/j.1365-3083.2007.02038.x

[31] J. J. McIntire , S. E. Umetsu , C. Macaubas , et al., “Hepatitis A Virus Link to Atopic Disease,” Nature 425 (2003): 576. 14534576 10.1038/425576a

[32] Z. Lu , Y. Yin , T. Rao , et al., “Interaction of Immune Cells With Renal Cancer Development: Mendelian Randomization (MR) Study,” BMC Cancer 24 (2024): 439. 38594655 10.1186/s12885-024-12196-8 PMC11005164

[33] S. K. Pal , R. Uzzo , J. A. Karam , et al., “Adjuvant Atezolizumab Versus Placebo for Patients With Renal Cell Carcinoma at Increased Risk of Recurrence Following Resection (IMmotion010): A Multicentre, Randomised, Double‐Blind, Phase 3 Trial,” Lancet 400 (2022): 1103–1116. 36099926 10.1016/S0140-6736(22)01658-0

[34] W. Xu , M. Puligandla , B. Halbert , et al., “Plasma KIM‐1 Is Associated With Recurrence Risk After Nephrectomy for Localized Renal Cell Carcinoma: A Trial of the ECOG‐ACRIN Research Group (E2805),” Clinical Cancer Research 27 (2021): 3397–3403. 33832947 10.1158/1078-0432.CCR-21-0025 PMC8287837

