# Supplementary material for: Kidney Injury Molecule‐1 Expression in Pathological T1b Clear Cell Renal Cell Carcinoma: A Putative Biomarker of High Immune‐Inflamed Status and Recurrence
Source: Pathol Int. 2025 May 14;75(7):340–8. doi: 10.1111/pin.70024 (PMC12288442; doi:10.1111/pin.70024)
Supplement: Supplementary file 1 — Supporting information. [file PIN-75-340-s001.docx]

Table s1. Relationship between cytoplasmic KIM-1 expression and clinicopathological parameters.

| Variables | Cytoplasmic KIM-1 expression | | *p* value |
| --- | --- | --- | --- |
|  | Low (n = 74) | High (n = 38) |  |
| Age (≥ 70/< 70) | 22/52 | 15/23 | 0.396 |
| Gender (male/female) | 52/22 | 30/8 | 0.374 |
| Tumor size (> 4 - < 5 cm/ ≥ 5 - ≤ 7 cm) | 37/37 | 14/24 | 0.231 |
| Laterality (left/right) | 38/36 | 21/17 | 0.842 |
| ECOG PS (0/ 1 and 2) | 63/11 | 33/5 | 1.000 |
| WHO/ISUP grade (1 and 2/ 3 and 4) | 35/39 | 9/29 | 0.024 |
| TAIC intensity 2 (%) | 12 (16) | 12 (32) | 0.088 |
| Hot TAIC status (%) | 11 (15) | 10 (26) | 0.200 |
| Tumor necrosis (%) | 13 (18) | 12 (32) | 0.100 |
| Lymphovascular invasion (%) | 32 (43) | 18 (47) | 0.693 |

ECOG PS, Eastern Cooperative Oncology Group Performance Status; KIM-1, kidney injury molecule-1; TAIC, tumor-associated immune cell; WHO/ISUP, World Health Organization/ International Society of Urological Pathology.
